# Soil Microbes Trade-Off Biogeochemical Cycling for Stress Tolerance Traits in Response to Year-Round Climate Change

**DOI:** 10.3389/fmicb.2020.00616

**Published:** 2020-05-13

**Authors:** Maria O. Garcia, Pamela H. Templer, Patrick O. Sorensen, Rebecca Sanders-DeMott, Peter M. Groffman, Jennifer M. Bhatnagar

**Affiliations:** ^1^Department of Biology, Boston University, Boston, MA, United States; ^2^Climate and Ecosystem Sciences Division, Lawrence Berkeley National Laboratory, Berkeley, CA, United States; ^3^Earth and Environmental Sciences, Lawrence Berkeley National Laboratory, Berkeley, CA, United States; ^4^Advanced Science Research Center at the Graduate Center, City University of New York, New York, NY, United States; ^5^Cary Institute of Ecosystem Studies, Millbrook, NY, United States

**Keywords:** climate change, forest ecology, microbial communities, soil freezing, warming, winter

## Abstract

Winter air temperatures are rising faster than summer air temperatures in high-latitude forests, increasing the frequency of soil freeze/thaw events in winter. To determine how climate warming and soil freeze/thaw cycles affect soil microbial communities and the ecosystem processes they drive, we leveraged the Climate Change across Seasons Experiment (CCASE) at the Hubbard Brook Experimental Forest in the northeastern United States, where replicate field plots receive one of three climate treatments: warming (+5°C above ambient in the growing season), warming in the growing season + winter freeze/thaw cycles (+5°C above ambient +4 freeze/thaw cycles during winter), and no treatment. Soil samples were taken from plots at six time points throughout the growing season and subjected to amplicon (rDNA) and metagenome sequencing. We found that soil fungal and bacterial community composition were affected by changes in soil temperature, where the taxonomic composition of microbial communities shifted more with the combination of growing-season warming and increased frequency of soil freeze/thaw cycles in winter than with warming alone. Warming increased the relative abundance of brown rot fungi and plant pathogens but decreased that of arbuscular mycorrhizal fungi, all of which recovered under combined growing-season warming and soil freeze/thaw cycles in winter. The abundance of animal parasites increased significantly under combined warming and freeze/thaw cycles. We also found that warming and soil freeze/thaw cycles suppressed bacterial taxa with the genetic potential for carbon (i.e., cellulose) decomposition and soil nitrogen cycling, such as N fixation and the final steps of denitrification. These new soil communities had higher genetic capacity for stress tolerance and lower genetic capacity to grow or reproduce, relative to the communities exposed to warming in the growing season alone. Our observations suggest that initial suppression of biogeochemical cycling with year-round climate change may be linked to the emergence of taxa that trade-off growth for stress tolerance traits.

## Introduction

High-latitude biomes such as boreal and temperate forests have experienced the fastest rates of climate warming of any ecosystem since the industrial revolution (Stocker et al., 2013) and future greenhouse gas emissions are expected to increase mean annual air temperatures at these locations in the coming century (Hoegh-Guldberg et al., 2018). Understanding how climate change may impact biogeochemical cycling in soils of high latitude forest ecosystems is critical to predicting global biogeochemistry in a warmer world, as these systems cover large geographic areas and hold ∼390 Pg of carbon (C) (Pan et al., 2011), as much as two-thirds of which is contained in soils (Dixon et al., 1994). However, climate warming produces contrasting temperature changes, which makes it difficult to predict biogeochemical cycling in high latitude, seasonally-snow covered ecosystems. Warming decreases soil temperatures in winter, as warmer air temperatures reduce snowpack and increase the extent and frequency of soil freeze/thaw events (Campbell et al., 2010; Brown and DeGaetano, 2011). Although warming during the snow-free times of year can initially increase C and nitrogen (N) fluxes as decomposition of soil organic matter increases (Rustad et al., 2001; Knorr et al., 2005; Brzostek and Finzi, 2011; Romero-Olivares et al., 2017), these effects can be reversed over the short term by increased frequency of soil freeze/thaw cycles in winter (Durán et al., 2014; Sorensen et al., 2016, 2018). Additional storage or loss of soil C and nutrients over the longer term will be determined by how the biogeochemical cycling activities of soil microorganisms (“effect traits”) are linked to traits that allow microbial species to persist under environmental stressors (“response traits”) (Allison and Martiny, 2008; Shade et al., 2012; Koide et al., 2014). Both warming and reduced winter snow cover can affect the species composition of soil microbial communities (Lipson and Schmidt, 2004; Allison and Treseder, 2008; Castro et al., 2010; Aanderud et al., 2013; Buckeridge et al., 2013; Luo et al., 2014; DeAngelis et al., 2015; Pold et al., 2015; Fernandez et al., 2016). However, it is unclear how shifts in species abundances under combined growing-season warming and soil freeze/thaw cycles in winter are connected to changes in ecosystem biogeochemistry and especially how species’ abilities to cycle C and N through soil is impacted by their tolerance for extreme fluctuations in environmental conditions. If we could link shifts in the taxonomic composition of soil microbial communities due to climate change in these regions with community-level physiology of the soil microbiome, we might be able to better anticipate future changes in forest C and nutrient cycling on regional or global scales.

Many soil microbial taxa are either resistant or resilient to climate change manipulations (Allison and Treseder, 2008; Allison et al., 2010; Waring and Hawkes, 2014; DeAngelis et al., 2015; Mucha et al., 2018), but soil C and N cycling may change if there is variation in the resistance or resilience of taxa within different groups or “functional guilds” of microbes that cycle C and N. For example, rising air temperatures are expected to reach optimal growth temperatures for some plant pathogens, which could increase pathogen loads and plant mortality, especially as pathogen mortality decreases and host density increases (Talbot, 2017). In addition, wood decay fungi, which deconstruct the cellulose and hemicellulose (i.e., brown rot fungi) and lignin (i.e., white rot fungi) components of dead plant material, are often more sensitive to changes in temperature than mycorrhizal fungi, which colonize live plant roots and exchange soil nutrients such as N and phosphorus (P) for C-rich photosynthate from their host plant (Diez et al., 2013; Boddy et al., 2014; Primicia et al., 2016; Gange et al., 2018). Decomposition activities of wood-decay fungi can increase with warming (A’Bear et al., 2014b; Crowther et al., 2015), which may account for often observed increases in soil carbon dioxide (CO_2_) flux following warming treatments (Luo et al., 2014; Xue et al., 2016). In addition, N-cycling processes associated with gaseous (nitrification and denitrification) and hydrologic losses of N are hypothesized to increase with more frequent soil freeze/thaw cycles that can disrupt plant-microbial interactions and increase inorganic N availability (Brooks et al., 2011; Morse et al., 2015). If this is the case, combined growing season soil warming and freeze/thaw cycles in winter could shift soil greenhouse gas emissions from CO_2_ to other trace gases, such as nitrous oxide (N_2_O). However, evidence for increased N_2_O flux under soil freeze/thaw is mixed; some studies have found that soil freeze/thaw cycles increase the flux of nitrous oxide (N_2_O) from soil (Groffman et al., 2006, 2011; Blankinship and Hart, 2012; Gao et al., 2018), while others have found the opposite (Reinmann et al., 2012). Furthermore, different microbial taxa often have individualistic responses to warming, even within functional groups (Sato et al., 2012; A’Bear et al., 2014a; Tatti et al., 2014; Venugopal et al., 2016). For example, different mycorrhizal fungi can respond positively or negatively to warming, depending on how warming impacts soil moisture, nutrient availability, and the physiology of their host plant (Rillig et al., 2002; Allison and Treseder, 2008; Hawkes et al., 2008; Compant et al., 2010; Fernandez et al., 2016; Wilson et al., 2016; Gange et al., 2018). Similar to warming responses, some microbial taxa can acclimate to freezing conditions, but different species within a trophic group (e.g., ectomycorrhizal fungi, arbuscular mycorrhizal fungi, and free-living saprotrophic decomposers) often differ in the level of tolerance to freezing (Addy et al., 1998; Klironomos et al., 2001; Robinson, 2001). Many functional groups (e.g., those involved in cellulose decomposition, nitrification and denitrification) include a wide taxonomic range of organisms, making it difficult to predict overall functional group responses to specific changes in climate.

An alternative way that microbial community shifts may be linked to changes in biogeochemical cycling with warming is through physiological trade-offs between growth or resource acquisition and survival mechanisms. Warming during the plant growing season can shift the composition of both soil fungal and bacterial communities initially toward fast-growing species (A’Bear et al., 2012) that have low carbon use efficiency (i.e., low biomass accrual, high CO_2_ respiration rates) (Frey et al., 2013; Li et al., 2019). These species often decompose cellulose and other labile plant polysaccharides as C resources (Melillo et al., 2002; Zhou et al., 2012; Luo et al., 2014; Pold et al., 2016) and can lead to an increase CO_2_ release from soil (Rustad et al., 2001; Romero-Olivares et al., 2017). However, growth and activity of belowground communities with warming is contingent on moisture availability, with microbial activity declining if soil moisture drops under rising temperatures (Allison and Treseder, 2008; Sorensen et al., 2018). The combination of growing-season warming and winter soil freeze/thaw cycles further suppresses biogeochemical cycling, reducing pools of extractable organic N and C in soils, as well as proteolytic and oxidative enzyme activity, microbial respiration, and microbial biomass N relative to growing-season warming alone (Sorensen et al., 2018). It’s unclear what changes in microbial communities under these conditions might lead to these changes in biogeochemical functions at the whole soil level. The compounded suppression of microbial activity under the more stressful conditions of winter soil freeze/thaw cycles may occur if active microbial biomass declines with freeze/thaw cycles, or if microbes trade-off decomposition traits (e.g., growth rate or enzyme activities) for traits that allow them to tolerate stressors such as desiccation, physical disturbances, and severe, fluctuating temperatures associated with freeze/thaw (Schimel et al., 2007; Malik et al., 2019). Such stress-tolerance traits may include the capacity to dehydrate (Klironomos et al., 2001); produce osmolytes (e.g., trehalose), thick cell walls (Schimel et al., 2007), anti-shock proteins (Robinson, 2001), or high numbers of multi-layer spores; survive anoxia (Hodkinson and Wookey, 1999); and form C-storage vesicles (Klironomos, 2000). Across microbial taxa, these traits tend to come at a cost to microbial biomass production (Crowther et al., 2014) and decomposition of plant structural polysaccharides (Treseder and Lennon, 2015), such that stress-tolerant taxa may be less directly active in the cycling of soil nutrients (Malik et al., 2019). Alternatively, taxa that persist under environmental stress may acclimate to these conditions by altering C allocation away from growth and toward stress tolerance mechanisms.

The aim of this study was to determine the combined effect of warming in the growing season, reduced winter snowpack, and increased frequency of soil freeze/thaw cycles on soil microbial communities and their capability to cycle C and N. We hypothesized that (1) a combination of growing season warming and increased frequency of soil freeze/thaw cycles in winter would lead to greater shifts in soil microbial communities than warming only. We expected that (2) these shifts would be primarily due to changes in the abundance of individual taxa within functional groups (e.g., arbuscular mycorrhizal fungi, denitrifying bacteria), rather than changes in the relative abundance of whole functional groups. We also hypothesized that (3) soil freeze/thaw events in winter may select for taxa that trade-off C decomposition traits and nutrient uptake traits for stress tolerance. To test our hypotheses, we sequenced both ribosomal DNA amplicons (ITS and 16S rDNA) and total microbial DNA (metagenomes) from soil microbial communities at the Climate Change across Seasons Experiment (CCASE) at the Hubbard Brook Experimental Forest (Templer et al., 2017). CCASE simulates warmer soils in the growing season and reduced snowpack in winter, inducing soil freeze/thaw cycles that allow us to examine the interacting effects of climate change across the year on soil communities and processes.

## Materials and Methods

### Site Description

Soil microbial communities were studied at CCASE at the Hubbard Brook Experimental Forest in the White Mountain National Forest in New Hampshire, United States (43° 56′N, 71°45′W; Templer et al., 2017). CCASE was set up in a ∼70–80-year-old forest stand, where canopy vegetation consists primarily of red maple (*Acer rubrum*; 64% basal area) and American Beech *(Fagus grandifolia*; 21% basal area). The Hubbard Brook Experimental Forest receives an average of 1400 mm of precipitation annually, 25–33% of which accumulates as snow (Bailey et al., 2003). Historically, mean air temperatures have been 19°C in July and −9°C in January and snowpack accumulates between mid-December until mid-April, with a peak depth in March of 1020–1270 mm (Campbell et al., 2010). Summers are short and cool, and winters are long and cold, with low temperatures that range between −12°C and −18°C, and shallow freezing in soils 2 out of 3 years (Campbell et al., 2010). Climate projections indicate that average annual air temperatures at Hubbard Brook will increase by approximately 2.9–5.3°C by the end of the century (Rustad et al., 2012) resulting in increased frequency of soil freeze/thaw cycles in winter (Campbell et al., 2010).

The Climate Change across Seasons Experiment consists of three climate change treatments applied across six stand-level plots (each 11 × 13.5m): two plots warmed +5°C above ambient soil temperature during the growing season (hereafter referred to as *warmed*), two plots warmed +5°C during the growing season and subject to soil freeze/thaw cycles in winter (hereafter referred to as *warmed* + *FTC*), and two reference plots (Templer et al., 2017; Sanders-DeMott et al., 2018). To warm plots, 4 km (2.5 mi) of heating cable were buried across the four climate treatment plots and turned on during the snow-free season (mid-April – mid-November) to maintain soil temperatures at +5°C above ambient. To induce soil freeze/thaw, snow is removed from the forest floor during winter by shoveling within 48 h of snowfall, which reduces soil temperature and induces soil freezing (Templer et al., 2012). Heating cables are then turned on after 72 h soil freezing events in winter to induce 72 h of soil thawing (Templer et al., 2017). Reference plots were disturbed to mimic cable installation, but do not have buried heating cables, because the disturbance from cable installation has minor impacts on soil biogeochemical processes or plant growth (Melillo et al., 2002). Heating cables were installed at CCASE in July 2013 and experimental treatments began in December 2013 with winter soil freeze/thaw cycles. Additional details of the CCASE experimental design can be found in Templer et al. (2017); Sanders-DeMott et al. (2018), and Sorensen et al. (2018).

### Soil Sample Collection

The upper soil organic horizon (3–10 cm soil depth) was collected from each plot at CCASE using a soil knife and a 10 × 10-cm frame. Each plot consists of four sampling quadrants, from which a single soil sample was collected at each of six time points throughout the growing season of 2014 [post-snowmelt (April 16), budburst (May 12), leaf-out (May 21), full canopy (June 1), peak growing season (July 22), and leaf senescence (September 24)]. This totaled 144 post-treatment soil samples (4 quadrant sub-replicates per plot × 3 treatments × 2 plots × 6 time points = 144 soil samples). Pre-treatment soil samples were also collected from each plot on July 1, 2013, before heating cables were turned on (3 treatments × 2 plots × 3 sub-replicates = 18 pre-treatment samples). All samples were stored on ice, transported back to the laboratory on the same day as sampling, and processed within 24 h. Soils were sieved through a 2 mm mesh sieve, rocks were removed, and soils homogenized by hand. Sieved soil was immediately flash frozen in liquid nitrogen and all samples were stored at −80°C prior to DNA extraction.

As part of previous work, biogeochemistry variables and environmental variables were measured on soils (Sanders-DeMott et al., 2018; Sorensen et al., 2018) or plants (Sanders-DeMott et al., 2018). Soils in each CCASE plot were analyzed for CO_2_ flux, net N mineralization, microbial biomass N, extractable organic C, dissolved inorganic N, total soil C and N, as well as soil temperature, moisture, and pH (Supplementary Table 1). These variables were measured in the lab at each post-treatment sampling time point on subsamples of the same soil cores taken for DNA extraction. Each variable was used in analysis of soil microbial community composition differences among CCASE treatments in the following growing season (2014, Supplementary Table 2). Other winter environmental variables were measured in each CCASE quadrant (e.g., maximum winter frost or snow depth, total frost or snow depth) or plot (minimum winter soil temperature at 10 cm, days with soil frost, number of freeze/thaw cycles) throughout the 2013/2014 (post-treatment) winter season. Then, a single value of these winter variables was used in analyses of soil microbial community composition at all time points in the following growing season (2014, Supplementary Table 2). Total frost or snow depth was calculated by integrating weekly measurements taken throughout the 2013/2014 winter season (Sanders-DeMott et al., 2018) into a single continuous variable (area under the curve, AUC; Sorensen et al., 2018), following Durán et al. (2014). For plants, root damage was measured (as relative electrolyte leakage, REL) on fine roots extracted from each soil sample at a single time point (post-snowmelt) in April 2014 (Sanders-DeMott et al., 2018). Photosynthesis rate was measured at a single time point in August 2014 and 2013 (peak plant biomass) on three leaves from each of four target red maple trees in each plot at 1000 umol PAR and 400 ppm CO_2_. Leaves were all attached to the same branch and were allowed to adjust for approximately 1–2 min until readings stabilized. Five measurements were taken at 15 s intervals over the course of 1 min. The average of the five measurements over 60 s was computed for each leaf to provide a single value for each of the measured leaves (*n* = 12 per plot: 3 leaves per tree × 4 trees). These values were then used to calculate average photosynthesis rate per plot as μmolCO_2_ m^–2^ s^–1^. Tree density as basal area was collected during peak biomass during the pre-treatment (2013) year (Templer et al., 2017; Sanders-DeMott et al., 2018). Changes in some biogeochemistry variables (photosynthesis rate, net N mineralization, microbial biomass N, total soil C and N, soil CO_2_ flux) could be calculated as the difference between pre- and post-treatment sampling date values.

### Microbial Community Analysis: DNA Amplicon Sequencing

DNA amplicons for taxonomic identification of fungi and bacteria were sequenced by the Department of Energy Joint Genome Institute (JGI; Walnut Creek, CA). Total DNA was extracted from each soil sample with the Powersoil DNA Extraction Kit (MoBio, Carlsbad, CA United States), cleaned with the PowerClean DNA Cleanup Kit (MoBio, Carlsbad, CA United States), and quality checked according to the JGI iTag Sample Amplification QC Standard Operating Procedure (iTagger v 1.1). Fungal and bacterial DNA was amplified at JGI following the JGI standard operating procedure for iTAG sequencing (Tremblay et al., 2015). Fungal DNA was amplified with the Illumina-adapted ITS9f/ITS4r primer pair that targets the ITS2 region of the rDNA operon (Ihrmark et al., 2012) and bacterial DNA was amplified with the 515f/806r primer pair that targets the V4 region of the 16S rDNA (Caporaso et al., 2012). 156 samples PCRed successfully (144 soil samples in 2014, 18 soil samples in 2013) and for these samples, both 16S and ITS amplicons were multiplexed on a single run on an Illumina MiSeq in 2 × 300 run mode at JGI. Sequencing yielded 31,295,994 ITS sequences and 27,340,707 16S sequences after quality checking (27,058 to 709,031 ITS sequences per sample and 66,874 to 329,664 16S sequences per sample). Raw sequence data is available at the Integrated Microbial Genomes with Microbiome Samples (IMG/M) online system (Chen et al., 2017).

### Metagenome Analysis: Shotgun DNA Sequencing

Shotgun metagenome sequencing was conducted for soil samples collected in May 2014 (full leaf-out), when soil biogeochemical differences among treatments were greatest (Sorensen et al., 2018). DNA extracts from each quadrant were pooled (4 per plot) to generate one metagenome library per plot (3 treatments × 2 plots = 6 metagenomes). Total genomic DNA was extracted by the same method as amplicon preparation. Library preparation was conducted at the JGI and sequenced on an Illumina HiSeq using standard JGI protocols (Huntemann et al., 2016). Sequencing yielded a total of 44.5 million read pairs across all samples (5.9 to 11 million reads per sample). Raw sequence data is available at the Integrated Microbial Genomes with Microbiome Samples (IMG/M) online system (Chen et al., 2017) and are referenced in the Genomes Online Database (GOLD Study ID Gs0114515). Each metagenome was assembled using the SPAdes 3.10.0-dev assembler (Bankevich et al., 2012). Assembly size for each metagenome ranged between 134,075,406 and 158,129,422 reads.

### Bioinformatics

Amplicon sequence data was analyzed using the iTagger 2.0 analysis pipeline (Tremblay et al., 2015) following the procedure of Caporaso et al. (2010). Briefly, FASTQ files were generated of each library, Duk^1^ was used to remove any contaminants (sequencing adapter dimers, human contaminants, etc.) and PCR primers of the conserved region were trimmed away. Chimeric sequences were detected using UCHIME^2^. This analysis yielded 31 million ITS sequences and 27 million 16S sequences after quality checking (41,000 to 615,000 ITS sequences per sample and 69,000 to 330,000 16S sequences per sample). High quality sequences were clustered into operational taxonomic units (OTUs) at 97% similarity (Glassman and Martiny, 2018), for both ITS and 16S, using USEARCH (Edgar, 2010). We omitted low-coverage soil samples (<16,000 usable reads) from the data set, resulting in amplicon data for a total of 127 soil samples used for downstream analyses.

Taxonomic classification of sequences was determined by matching representative sequences for an OTU against the UNITE database for fungi (Kõljalg et al., 2013) or the Greengenes database for bacteria (DeSantis et al., 2006), using the QIIME implementation of the RDP Classifier (Wang et al., 2007). All non-fungal samples were discarded from the ITS dataset and non-bacterial sequences were discarded from the 16S dataset. The final ITS dataset for analysis was comprised of 7177 taxa, 85% of which were fungal. The final 16S dataset for analysis was comprised of 12055 taxa, 93% of which were bacterial. Functional guilds were assigned to both bacteria and fungi at the genus level. Fungal OTUs were categorized into functional guilds using the FunGuild classification for fungal genera (Nguyen et al., 2016). This assignment method resulted in a functional guild classification for 65% of fungal taxa. Bacterial OTUs were categorized into functional guilds based on the presence of genes within bacterial genomes that coded for proteins in the complete pathways for N fixation, denitrification, and cellulose degradation (Berlemont and Martiny, 2013; Berlemont et al., 2014; Albright et al., 2018). For N cycling, bacterial genera with all pathways present in their genomes were classified as “N fixers” or “denitrifiers.” For C cycling, bacteria with at least one of the genes coding for cellulose-degrading enzymes (listed below) were classified as “cellulose decomposers.” Bacterial functional guilds were supplemented with information from the CCASE soil metagenomes about the genera known to possess genes within their genomes that coded for proteins responsible for specific enzymatic reactions within each C and N cycling pathway. These genes included nitrogenase for N fixers, nitrate reductase (*nar* G, H, or I), nitrite reductase (*nir* K, S), nitric oxide reductase (*nor* B, C), and nitrous oxide reductase (*nos* Z) for denitrifiers; β-glucosidase (GH 1, 3) and cellulases (GH 5, 6, 7, 8, 9, 12, 44, 45, 48) for cellulose decomposers. We searched our CCASE soil metagenomes for the taxa possessing these genes within their genomes (described below). Collectively, we identified 28 bacterial genera in our dataset as “N fixers,” 13 genera as “denitrifiers,” and 173 genera as “cellulose decomposers.” This assignment method resulted in a functional guild classification for 9% of bacterial taxa. In addition, bacterial phyla were classified as “copiotrophic” or “oligotrophic” based on ecological classification of phyla from the literature (Fierer et al., 2007, 2011).

To estimate fungal diversity in each sample, we calculated within-sample diversity (alpha diversity) as observed OTU diversity estimates for each soil sample rarefied to 16,000 sequences/sample (for fungi) and 40,000 sequences/sample (for bacteria) using the alpha_rarefaction.py command in QIIME (Caporaso et al., 2010). To estimate microbial community dissimilarity among samples, we calculated across-sample diversity (beta-diversity) as the quantitative Bray-Curtis dissimilarity metric, using the beta_diversity.py command in QIIME. Prior to calculating beta-diversity among samples, fungal OTU counts were normalized via rarefaction to 16,000 sequences, and prokaryotic OTU counts were rarefied to 40,000 sequences. Rarefaction was used to normalize sequences because nearly all samples had good sequence coverage of OTUs, but samples varied more than 10-fold in sequencing depth (Weiss et al., 2016).

Soil metagenome data was analyzed by the standard JGI metagenome analysis pipeline (Huntemann et al., 2016). Briefly, assembled and unassembled reads were trimmed to remove low-quality regions and sequences shorter than 150 bp were removed. Quality-filtered sequences were then categorized as ribosomal RNA genes (5S, 16S, and 23S) using the hmmsearch tool from the package HMMER 3.1b2, or as protein-coding genes using a consensus of four gene prediction tools: prokaryotic GeneMark.hmm (v. 2.8), MetaGeneAnnotator (v. Aug 2008), Prodigal (v. 2.6.2) and FragGeneScan (v. 1.16). Ribosomal RNA genes were identified using curated models generated by JGI from full length genes within the IMG/M system (Markowitz et al., 2014). Gene functions were obtained by downloading Pfam domain annotations for each metagenome and by searching each metagenome in the IMG system for gene product (Huntemann et al., 2016). Raw annotation counts for each Pfam domain or gene product name were downloaded directly for each metagenome sample from the IMG site, then normalized as read counts per 10,000 genes (Knight et al., 2018). This normalization procedure produced qualitatively similar results to other recommended normalization approaches (e.g., TMM normalization, normalization to the single-copy gene RecA; Tang et al., 2013; Pereira et al., 2018) for discerning differentially abundant genes across treatments. For each soil metagenome, we downloaded counts of all Pfam domains, as well as counts of genes hypothesized to be involved in stress response (1,3-β-glucan synthase, trehalase, RNA helicase, oxidases involved in melanin synthesis; Treseder and Lennon, 2015) or carbon (cellulose) use (β-glucosidase, cellobiosidase, endoglucanase, cellulase) and nitrogen use (e.g., nitrogenase, nitrite reductase, nitric oxide reductase, nitrous oxide reductase) by soil microbes (Supplementary Table 3). Bacterial taxonomic assignments for each functional gene within the soil metagenome were extracted by searching the CCASE soil metagenome samples by gene product name in IMG and downloading the taxonomic ID associated with each gene scaffold.

### Statistics

#### General Statistical Approach

All statistical tests and graphics were done in R version 3.1.3 (R Team, 2013), with graphics plotted using the “ggplot2” package (Wickham, 2009). Bacterial and fungal data were analyzed separately. Measurements of some environmental variables (maximum snow or frost depth, tree basal area, photosynthesis rate, minimum winter soil temperature, days with soil frost, and number of freeze/thaw cycles) made within each CCASE plot were averaged to obtain one plot mean for each measurement type on each sampling date (Supplementary Table 1). The plot mean was then used in each statistical analysis using these factors. By contrast, measurements of soil chemistry (i.e., soil% C, soil% N, soil pH, soil moisture, extractable organic C, amino acid-N), certain aspects of microbial activity in soil (i.e., net N mineralization rates, microbial biomass N, extracellular enzyme activities), and other soil environmental variables (soil temperature at time of sampling, root relative electrolyte leakage) were collected within each quadrant within each plot (Sanders-DeMott et al., 2018; Sorensen et al., 2018) and analyzed as such for correlating with changes in microbial communities.

Linear mixed-effect models were used to determine the effect of the experimental climate treatments (i.e., fixed-effect) on soil microbial communities. Models were run using the “vegan” package in R (Oksanen et al., 2011). Regression models were run using CCASE treatment, sampling date, and/or environmental and soil variables measured previously (Supplementary Tables 4, 5) as the independent variable, depending on the analysis (described below). In cases where samples from each quadrant per plot were assayed separately, we included quadrant nested within plot as a random effect in each model, to account for non-independence of nested sampling (Sanders-DeMott et al., 2018). *Post hoc* pairwise comparisons among groups were obtained using the “HSD.test” command in the “agricolae” package (Mendiburu Delgado, 2009) in R. In cases where data did not conform to assumptions of normality and homogeneity of variance, values were log or rank transformed prior to analysis. All statistical tests were considered significant at *P* < 0.05.

#### Statistical Tests Used to Assess Climate Treatment Effects

To determine the effect of climate treatments on alpha diversity, ANOVAs were used with CCASE treatment crossed with sampling date as the fixed independent variable, sampling quadrant nested within plot as the random variable, and observed microbial richness as the dependent variable. To determine the effect of climate treatments on microbial community composition, permutational MANOVA (i.e., PERMANOVA) tests were performed with Bray-curtis dissimilarity in community composition among samples (for either fungi or bacteria) as the dependent variable using the “adonis” function in the “vegan” package in R (Oksanen et al., 2011). Single-factor regression models were run first with CCASE treatment, sampling date, or environmental or soil factors and sampling quadrant or plot as the independent variables. To determine the specific environmental factors that correlated with microbial community shifts, we then conducted multiple regression on matrices (MRM) using those environmental or soil factors that showed significant correlation with fungal or bacterial community metrics in univariate analyses. Due to strong correlations between many of our environmental variables (Supplementary Tables 4, 5), we included only those variables that explained over 2% of variation in community dissimilarity in univariate analyses (Talbot et al., 2014). MRM was performed with the package “Ecodist” (Goslee and Urban, 2007). Non-metric multidimensional scaling (NMDS) was used to provide a graphical representation of relationships between communities across samples (McCune et al., 2002).

To determine how taxa and functional groups responded to climate change treatments, we calculated the change in relative abundance of groups of fungi or bacteria in soils of each plot after CCASE treatments were applied (in the growing season of 2014) relative to pre-treatment (July 2013). We report community shifts as a change in relative abundance of taxa from pre-treatment to post-treatment to account for confounding effects of spatial autocorrelation on community composition, as well as to leverage the relatively rare pre-treatment microbiome data from CCASE. The presence of pre-treatment measurements at CCASE is unique, as most climate change manipulation experiments are established prior to sampling for soil microbial communities. Our approach provides a relatively conservative test of treatment effects on microbial communities, because it accounts for variability in taxon relative abundances in both space and time. Relative abundance of microbial taxa and functional groups (e.g., arbuscular mycorrhizal) was calculated by summing the relative abundance of individual OTUs within a genus, phylum, or functional group in a plot at each individual time point during the 2014 growing season, then calculating the difference between pre-treatment (2013) and post-treatment (2014) time points. To determine the effect of climate treatments on changes in taxon and functional group relative abundances, factorial ANOVA was used with the change in relative abundance of genus, phylum, or functional group of fungi or bacteria in soil as the dependent variable. The interaction between sampling date and CCASE treatment was the fixed effect and plot was the random effect. To determine the relationship between changes in microbial group abundances and biogeochemical processes, we used linear regression with change in relative abundance of a functional group (e.g., cellulose decomposers) as the independent variable and change in a plant or soil process (e.g., photosynthesis rate at peak plant biomass, rate of net soil N mineralization, microbial biomass N, total soil C or N concentration, soil CO_2_ flux) from pre-treatment or extracellular enzyme activities as the dependent variables. Changes in process rates were correlated to changes in functional groups measured at that same time point.

To determine relationships between metagenome content of soils and climate treatments, we conducted two analyses. First, we conducted single factor ANOVA with the normalized relative abundance of a gene of interest (Supplementary Table 3) as the dependent variable and CCASE treatment as the independent variable. Second, we tested for a global association between all Pfam domains within each metagenome and CCASE treatment using the “signassoc” function in the “indicspecies” package in R (Cáceres and Legendre, 2009). We tested whether or not the frequency of Pfam domains in each climate treatment was significantly higher (gene gains) or lower (gene losses) than random using a corrected alpha of 0.1, due to the small number of replicates per treatment (Sistla et al., 2013; Pold et al., 2016). Biochemical pathway enrichment analysis was conducted for Pfam domains that were significantly positively or negatively associated with climate treatments using dcGO (Fang and Gough, 2013). Biochemical pathways were assigned as gene ontology (GO) terms, using an adjusted FDR threshold of 0.1 and the full set of GO terms for Pfam domains associated with each climate treatment. Results from dcGO were visualized in REVIGO (Supek et al., 2011).

## Results

### Microbial Community Changes With Climate Treatments

**FIGURE 1:**
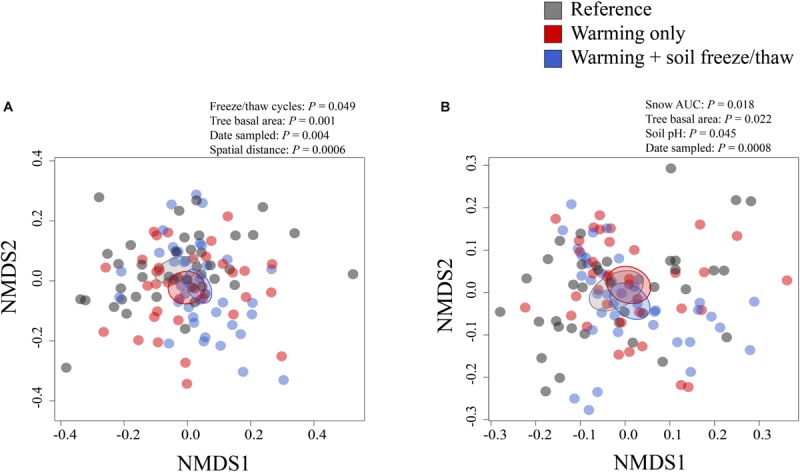
Shifts in soil **(A)** fungal and **(B)** bacterial community composition with climate change treatments at CCASE. Samples are colored by climate treatments. Statistics are derived from multiple regression (MRM) analysis (Supplementary Table 2b). Points represent individual soil samples sequenced from each quadrant in each plot. Ellipsoids represent the 95% confidence interval around the centroid for each climate treatment group.

Different climate change treatments at CCASE harbored unique bacterial and fungal communities in soils (Figure 1). Across sampling time points, soil bacterial community composition correlated most strongly with total snow depth and fungal community composition correlated most strongly with soil freeze/thaw cycles in the previous winter (Supplementary Table 2b). Bacterial and fungal communities also varied by other environmental factors, including total tree basal area, sampling date, and spatial distance between samples (in the case of fungi) or soil pH (in the case of bacteria).

While the composition of microbial communities changed across CCASE treatments, the richness of bacterial and fungal taxa in soils did not differ between any of the treatments (Supplementary Figure 1). In addition, microbial community composition changes with treatment were relatively minor, with 18–36% of the soil community unique to the *warmed* treatment and 20–32% of the soil community unique to *warmed* + *FTC* treatment. Across CCASE treatments, shifts in soil fungal communities with CCASE treatments were consistently greater than shifts in the bacterial communities (Supplementary Figure 2). Changes in the relative abundance of fungi in the phylum Ascomycota under the *warmed* + *FTC* treatment relative to pre-treatment soils were significantly lower than that of fungi in the phylum Basidiomycota in soils (Supplementary Figure 3). Changes in the relative abundance of bacterial taxa in the phyla Actinobacteria, Bacteriodetes, and Proteobacteria under the *warmed* + *FTC* treatment relative to pre-treatment were also lower than under reference or *warmed* treatment, while bacteria in the Chloroflexi, Firmicutes, and Verrucomicrobia were higher (Supplementary Figure 4).

### Microbial Functional Shifts With Climate Treatments

**FIGURE 2:**
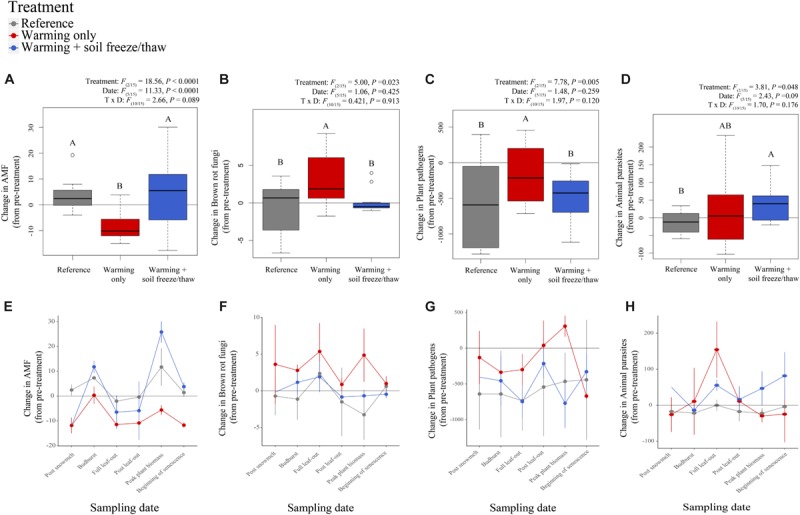
Dynamics of fungal functional guilds in soil at CCASE. Functional guilds include arbuscular mycorrhizal fungi (AMF) **(A,E)**, brown rot fungi **(B,F)**, plant pathogens **(C,G)**, and animal parasites **(D,H)**.

Soil fungal and bacterial taxa that changed in relative abundance with climate treatments belonged to specific functional groups that have unique roles in biogeochemical cycling. On average across sampling time points, arbuscular mycorrhizal fungi decreased significantly in relative abundance under the *warmed* treatment, yet recovered under the *warmed* + *FTC* treatment, relative to pre-treatment soils (Figure 2). While arbuscular mycorrhizal fungi were generally reduced in the *warmed* treatment plots relative to reference plots before warming treatments commenced in the growing season (April), differences between the *warmed* treatment and the *warmed* + *FTC* treatment increased throughout the growing season (Figure 2). In addition, both plant pathogenic fungi and brown rot fungi increased in relative abundance under the *warmed* treatment, but returned to pre-treatment abundances in *warmed* + *FTC* plots (Figure 2). Fungal parasites of animals increased significantly in *warmed* + *FTC* plots relative to reference plots (Figure 2).

**FIGURE 3:**
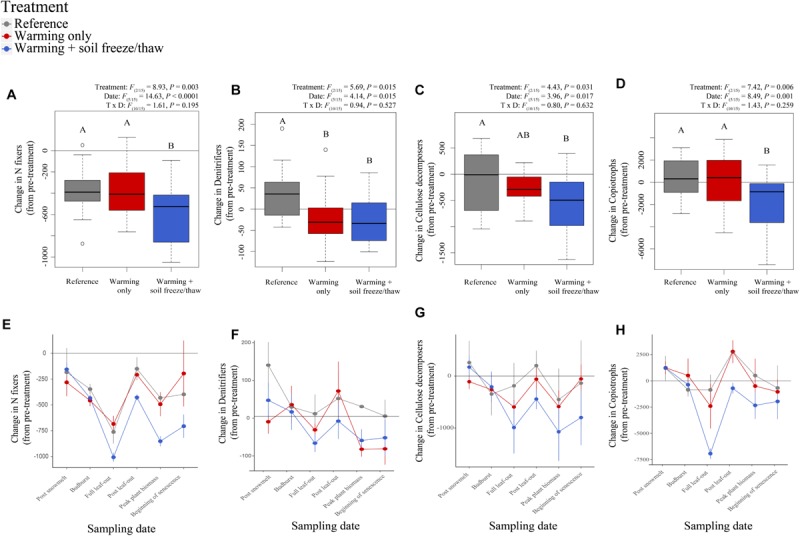
Dynamics of bacterial C and N cycling functional guilds in soil at CCASE. N-fixing bacteria were categorized as those with the genetic capacity to produce nitrogenase **(A,E)**, denitrifying bacteria **(B,F)** were categorized based on the presence of genes involved in complete oxidation/reduction of N from NO_3_^–^ to N_2_ [i.e., nitrate reductase (*nar* G, H, or I), nitrite reductase (*nir* K, S), nitric oxide reductase (*nor* B, C), nitrous oxide reductase (*nos* Z)], and cellulolytic bacteria **(C,G)** were categorized as those with the genetic capacity to generate either extracellular β-glucosidase (GH 1, 3) and/or cellulases (GH 5, 6, 7, 8, 9, 12, 44, 45, 48). Copiotrophic bacteria **(D,H)** were categorized based on ecological classification of phyla from the literature.

Within bacterial communities, N-fixing taxa in soil declined under changes in soil temperature relative to pre-treatment soils, declining significantly more under the *warmed* + *FTC* treatment relative to reference soils and *warmed* soils (Figure 3). Similarly, at full-leaf out, the relative abundance of genes encoding nitrogenase (for N fixation) was significantly lower under the *warmed* + *FTC* treatment relative to reference and *warmed* soils (Supplementary Figure 5).

The relative abundance of denitrifying bacteria (i.e., those that reduce nitrite (NO_2_^–^) to nitric oxide (NO), NO to nitrous oxide (N_2_O), and N_2_O to N_2_) in soil also significantly decreased under the *warmed* + *FTC* treatment (Figure 3). Within the soil metagenome, genes encoding N_2_O reductase (that catalyzes the final step in denitrification, reducing N_2_O to N_2_) also correlated positively with total snow depth [*t*(4) = 2.83, *F*_(1/4)_ = 7.99, *P* = 0.048], although genes coding for NO reductase did not [*t*(4) = −0.55, *F*_(1/4)_ = 0.30, *P* = 0.613]. In fact, the ratio of NO reductase to N_2_O reductase genes detected at full leaf-out declined significantly with increasing minimum soil temperature experienced the prior winter (Supplementary Figure 6) due to declines in N_2_O reductase genes. No other bacterial functional groups or genes associated with N cycling in soil (e.g., dissimilatory nitrate reduction, nitrification) changed significantly with climate treatments. However, genes coding for ammonium transporters increased in relative abundance with increasing minimum soil temperature in winter [*t*(4) = 3.21, *F*_(1/4)_ = 10.33, *P* = 0.033; Supplementary Figure 5].

C-cycling bacteria in soil also shifted with climate treatments. Both cellulolytic bacteria and those bacteria that are typically considered copiotrophic (bacteria in the phyla Bacteriodetes, Proteobacteria, and Actinobacteria) declined significantly under the *warmed* + *FTC* treatment across time points, relative to pre-treatment soils (Figure 3). Genes coding for cellulose breakdown (cellulase and β-glucosidase) detected at full leaf-out were also significantly lower in soils under the *warmed* + *FTC* treatment. This effect was related primarily to differences in total snowpack between sites, with cellulase and β-glucosidase genes correlating positively with total snow AUC and negatively with frost AUC (Supplementary Figure 5).

Changes in the relative abundance of fungal and bacterial functional groups in soil with climate treatments were driven by shifts in the relative abundance of dominant genera within those groups. Changes in arbuscular mycorrhizal fungi were determined primarily by species in the genus *Glomus*, the dominant arbuscular mycorrhizal genus at CCASE, which declined significantly in soils under the *warmed* treatment and recovered under the *warmed* + *FTC* treatment (Supplementary Figure 7). Changes in the relative abundance of brown rot fungi in soil with warming were driven by increases in the dominant brown rot genera *Cerinosterus* and *Amylocystis*. Increases in animal parasites under the *warmed* + *FTC* treatment were related to increases in the genera *Trichosporon* and *Metarhizium*. For bacteria, changes in the relative abundance of N fixers and cellulose decomposers in soil were driven by shifts in the genus *Burkholderia*, which dropped significantly under the *warmed* + *FTC* treatment after full leaf-out during the following year (Supplementary Figure 7).

**FIGURE 4:**
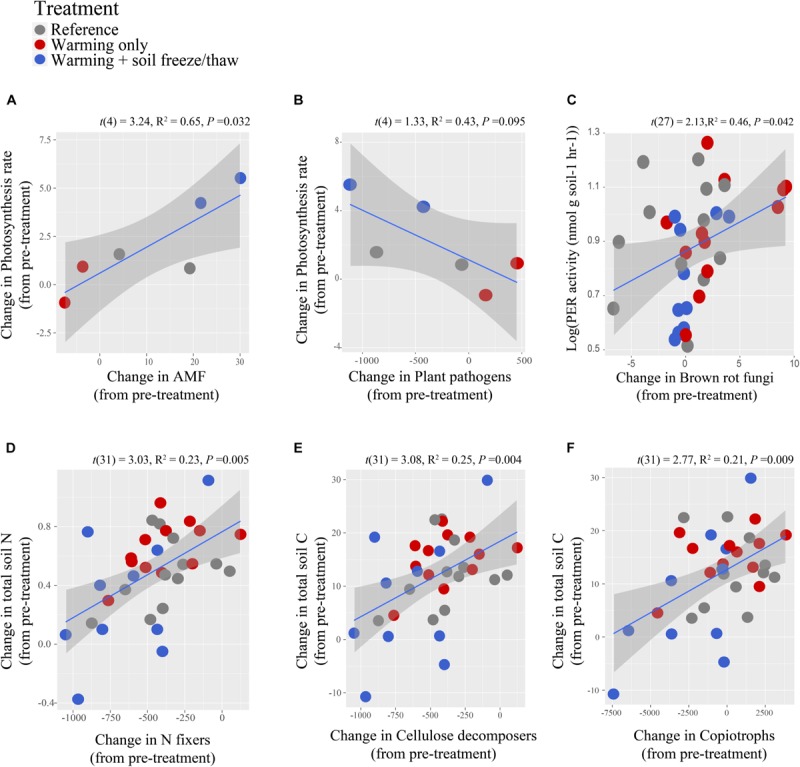
Relationship between microbial C and N cycling functional guilds and biogeochemical processes in soil at CCASE. Change in relative abundance of arbuscular mycorrhizal fungi (AMF) **(A)**, plant pathogenic fungi **(B)**, brown rot fungi **(C)**, N-fixing bacteria **(D)**, cellulolytic bacteria **(E)** and copiotrophic bacteria **(F)** each correlated with at least one metric of C and N cycling processes in soil. Changes in functional group relative abundance and biogeochemistry processes were calculated relative to pre-treatment (2013) values averaged at the plot-level. Changes in process rates were correlated to changes in functional groups measured at that same time point.

Changes in arbuscular mycorrhizal abundance correlated negatively with changes in N mineralization throughout the year, relative to pre-treatment rates (*R*^2^ = 0.46, *t*(21) = −2.11, *F*_(6/21)_ = 4.81, *P* = 0.047) and correlated positively with changes in the rate of photosynthesis at peak growing season (Figure 4, *R*^2^ = 0.65, *t*(4) = 3.24, *F*_(1/4)_ = 10.47, *P* = 0.032). Changes in plant pathogens tended to correlate negatively with changes in the rate of photosynthesis at peak growing season (*R*^2^ = 0.43, *t*(4) = −2.17, *F*_(1/4)_ = 4.73, *P* = 0.095). Changes in brown rot relative abundance correlated positively with potential peroxidase activity in these soils (*R*^2^ = 0.46, *t*(27) = 2.13, *F*_(6/27)_ = 5.69, *P* = 0.042). Changes in the relative abundance of N fixers correlated positively with changes in total soil N relative to pre-treatment (Figure 4, *R*^2^ = 0.23, *t*(33) = 3.03, *F*_(1/33)_ = 5.98, *P* = 0.005), as well as activity of acid phosphatase (*R*^2^ = 0.36, *t*(33) = 2.26, *F*_(1/33)_ = 10.45, *P* = 0.03) in soil. Changes in C-cycling bacterial groups in soil correlated positively with soil C cycling metrics, including cellobiohydrolase activity (cellulolytic: *R*^2^ = 0.11, *t*(33) = 2.23, *F*_(1/33)_ = 3.10, *P* = 0.033; copiotrophic: *R*^2^ = 0.26, *t*(33) = 3.52, *F*_(1/33)_ = 6.94, *P* = 0.001), beta-glucosidase activity [cellulolytic: *R*^2^ = 0.17, *t*(33) = 2.76, *F*_(1/33)_ = 4.50, *P* = 0.010; copiotrophic: *R*^2^ = 0.32, *t*(33) = 4.02, *F*_(1/32)_ = 8.90, *P* = 0.0003], changes in rates of soil respiration [cellulolytic: *R*^2^ = 0.22, *t*(30) = 2.66, *F*_(2/30)_ = 5.46, *P* = 0.012; copiotrophic: *R*^2^ = 0.24, *t*(30) = 2.82, *F*_(2/30)_ = 5.92, *P* = 0.009], and changes in total soil C concentrations [Figure 4, cellulolytic: *R*^2^ = 0.25, *t*(31) = 2.77, *F*_(2/31)_ = 6.37, *P* = 0.009; copiotrophic: *R*^2^ = 0.21, *t*(31) = 3.08, *F*_(2/31)_ = 5.39, *P* = 0.004] relative to pre-treatment, such that these activities declined under the *warmed* + *FTC* treatment. Declines in cellulolytic and copiotrophic bacteria also correlated with changes in total soil N concentrations [cellulolytic: *R*^2^ = 0.25, *t*(31) = 3.21, *F*_(2/31)_ = 6.61, *P* = 0.004; copiotrophic: *R*^2^ = 0.24, *t* (31) = 3.10, *F*_(2/31)_ = 6.22, *P* = 0.005].

### Relationship Between Stress-Tolerance Traits and Climate Treatments

**FIGURE 5:**
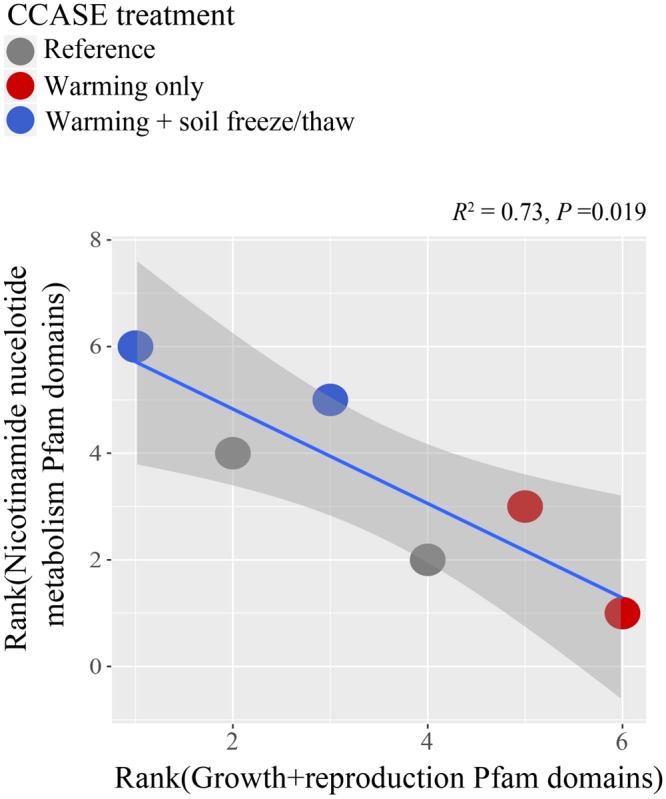
Correlation between NADH/NAD^+^ metabolism genes and genes associated with growth and reproduction in the soil metagenome at full leaf-out.

Climate change treatments across seasons shifted the genetic potential for stress tolerance within soil microbial communities. At the time of full leaf-out, microbial genes coding for tyrosinase (involved in melanin synthesis) were significantly lower in soils under the *warmed* treatment relative to under the *warmed* + *FTC* treatment [*F*_(2/3)_ = 19.60, *P* = 0.019]. However, there was no effect of climate treatment on other genes hypothesized to confer tolerance to extreme environmental conditions, including genes coding for 1,3-β-glucanase [*F*_(2/3)_ = 0.29, *P* = 0.768], trehalase [*F*_(2/3)_ = 0.59, *P* = 0.609], or RNA helicase [*F*_(2/3)_ = 1.50, *P* = 0.354]. Instead, soil microbes in the *warmed* treatment had high abundances of genes involved in stress-activated signaling cascades in the cell (JNK cascade), as well as genes coding for proteins involved in endocytosis/cell transport, cyclic compound metabolism, reproduction, and growth (Supplementary Figure 8). By contrast, soils in the *warmed* + *FTC* treatment had significantly higher abundances of genes coding for proteins involved in oxidation and reduction reactions in the cell, specifically nicotinamide nucleotide coenzyme (NADH/NAD^+^) metabolism, compared to both reference soils and the *warmed* treatment soils (Supplementary Figure 8). Across CCASE treatments, NADH/NAD^+^ metabolism genes in the soil metagenome were negatively correlated with genes associated with growth and reproduction (Figure 5). A handful of genes also declined significantly with climate treatments. Genes coding for proteins involved in purine compound metabolism, as well as genes involved in organic compound biosynthesis and co-factor metabolism, declined in relative abundance under the *warmed* treatment. By contrast, genes coding for proteins involved in cell differentiation (e.g., lateral inhibition) declined in relative abundance under the *warmed* + *FTC* treatment (Supplementary Figure 8).

## Discussion

Temperatures in high latitude forest ecosystems have been increasing approximately twice as fast as the global mean during the last century and are projected to warm a further 3–8°C by 2100 (Hartmann et al., 2013). Cascading impacts of warming on winter snowpack are expected to increase the frequency and severity of soil freeze/thaw cycles in winter (Hayhoe et al., 2007; Campbell et al., 2010; Brown and DeGaetano, 2011), prompting us to ask, “How do severe soil temperature fluctuations in winter interact with the effects of warming during the growing season to impact soil microorganisms?” The answer depends on how microbial activity is linked to the persistence, loss, or altered physiology of taxa that inhabit soils experiencing these stressful climatic conditions. We hypothesized that soil freeze/thaw cycles in winter would lead to greater shifts in belowground microbial communities than warming in the growing season only. In line with this hypothesis, we found that climate change across seasons (i.e., combined warming during the growing season and soil freeze/thaw cycles in winter, the *warmed* + *FTC* treatment) shifted the composition of belowground fungal and bacterial communities more so than warming during the growing season alone (the *warmed* treatment, Figure 1). We also hypothesized a trade-off in C and N cycling metabolism with stress tolerance, following previous observations that soil C and N cycling slowed under winter climate change (Sorensen et al., 2018). In line with this hypothesis, we found that the *warmed* + *FTC* treatment suppressed both microbial taxa with strong C decomposition traits and taxa involved in key parts of the soil N cycle (Figures 2–4 and Supplementary Figure 5). We also found a trade-off between some of the genetic capacity for soil bacterial community members to grow or reproduce and to generate intracellular redox activity in response to the stress of extreme climate fluctuations (Figure 5). Our results indicate that winter climate change reshapes the soil microbial community beyond the impact of growing-season warming alone, promoting the emergence of stress-tolerant taxa that could have long-term impacts on C and N cycling in forest soils.

### Hypothesis 1: Year-Round Climate Change Shifted Specific Lineages of Soil Microbes

We found that fungal and bacterial communities in soil responded to climate change manipulations in the field, similar to previous work (Lipson and Schmidt, 2004; Allison and Treseder, 2008; Castro et al., 2010; Aanderud et al., 2013; Buckeridge et al., 2013; Luo et al., 2014; DeAngelis et al., 2015; Pold et al., 2015; Fernandez et al., 2016; Wertz et al., 2016). The microbial taxa that turned over with CCASE treatments represented a small fraction of both bacterial and fungal communities, yet were high in number (Supplementary Figure 2), perhaps because of the high read counts per sample we obtained through sequencing. While other studies have also noted impacts of either growing-season warming or reduced snowpack on soil microbial diversity (Zinger et al., 2009; DeAngelis et al., 2015), our treatments at CCASE had no impact on soil fungal or bacterial diversity metrics. These results are consistent with others that have noted stronger response of microbial taxonomic composition than taxonomic richness to changes in soil temperature (DeAngelis et al., 2015).

Fungi tended to be more sensitive to CCASE treatments than bacteria (Supplementary Figure 2), which may be due to different climate tolerances of fungi and bacteria (Buckeridge et al., 2013) or generally finer levels of phylogenetic resolution of the rDNA region sequenced for fungi (genus-level) compared to bacteria (family-level). However, both fungi and bacteria were sensitive to CCASE treatments at coarse taxonomic scales as well. Fungi in the phylum Ascomycota did not respond to the *warmed* treatment (Supplementary Figure 3), in line with reports from other field-based warming experiments (Allison and Treseder, 2008; Geml et al., 2015; Morgado et al., 2015; Fernandez et al., 2016). While this pattern may reflect the general tolerance of Ascomycetes to warming-induced desiccation stress in soil (Gehring et al., 1998), in our study, Ascomycetes did not appear to track soil moisture. We hypothesize that the resistance of Ascomycetes to growing-season warming is related to their generally faster growth and reproduction rates and copiotrophic lifestyle relative to Basidiomycetes (Webster and Weber, 2007). By contrast, fungi in the phylum Basidiomycota were higher in relative abundance under the *warmed* + *FTC* treatment compared to Ascomycetes (Supplementary Figure 3). In addition, we found that the bacterial phyla Actinobacteria, Bacteriodetes, and Proteobacteria tended to decline under the *warmed* + *FTC* treatment, while bacteria in the Chloroflexi, Firmicutes, and Verrucomicrobia tended to increase under those conditions (Supplementary Figure 4). These phylum-level dynamics differ from those observed in studies that only warmed soils throughout the year (DeAngelis et al., 2015) or removed snow in winter to induce soil freeze/thaw (Aanderud et al., 2013). These differences could be because (1) organisms in these lineages have low tolerance for the environmental variations experienced year-round in the *warmed* + *FTC* treatment, such as increased salt levels in the unfrozen water within mostly frozen soils or physical disruption of habitat associated with frost heaving, and/or (2) the taxa within those phyla that responded to CCASE treatments differ from those responding to climate manipulations in other systems.

### Hypothesis 2: Year-Round Climate Change Shifted Microbial Functional Groups in Soil

Whole functional groups of soil microorganisms shifted in response to CCASE treatments, yet these shifts were driven by few taxa within each group, rather than consistent, directional shifts in many taxa comprising the group (Supplementary Figure 7). For example, the *warmed* treatment favored brown rot fungi involved in carbohydrate decomposition and metabolism (Figure 2), consistent with previous observations that warming can stimulate breakdown and respiration of labile soil C in the short term (Zhou et al., 2012; Luo et al., 2014; Pold et al., 2016; Xue et al., 2016). Increases in brown rot fungi within *warmed* treatment plots were primarily due to increases in the genus *Cerinosterus*, as was the decline of brown rots under the *warmed* + *FTC* treatment. These results are consistent with others who have found that environmental stressors can select for new taxa in soil microbial communities, even if total abundance of a functional group remains the same (Tatti et al., 2014). Bacterial groups involved in C cycling did not change significantly with growing-season warming alone; instead, both copiotrophic bacteria and cellulose-degrading bacterial genera declined significantly under the *warmed* + *FTC* treatment (Figure 3). Cellulose-degrading enzyme activity and changes in the concentrations of soil C and N following CCASE treatments correlated with changes in both copiotrophs and cellulolytic bacteria (Figure 4), but not with changes in brown rot fungi, suggesting that brown rots may persist under warming due to the ability of this group to tolerate high summer temperatures and lower soil moisture conditions compared to other microbial groups (Carll and Highley, 1999), rather than their C decomposition capabilities.

Growing-season warming favored the emergence of plant pathogens detected in soil, while combined warming and soil freeze/thaw cycles increased the relative abundance of animal parasites (Figure 2). Persistence of these guilds under simulated climate changes may be due to key stress-tolerance mechanisms that also confer pathogenicity. For example, melanin protects fungal cells from temperature and freeze/thaw stress while also increasing infectivity of many fungal pathogens (Talbot, 2017), such that global warming is expected to increase the relative abundance of melanized fungi in soil (Nevo, 2012). Changes in photosynthetic rates of dominant trees at CCASE correlated positively with changes in the relative abundance of arbuscular mycorrhizal fungi, which was lower under the *warmed* treatment than under the *warmed* + *FTC* treatment (Figure 4). The decline of arbuscular mycorrhizal fungi under the *warmed* treatment relative to the *warmed* + *FTC* treatment closely tracked shifts in the genus *Glomus* (Supplementary Figure 7). Correlations between changes in arbuscular mycorrhizal fungi and changes in photosynthesis rates with CCASE treatment suggest that plant growth may be driven by the activity of this individual genus, rather than the coordinated activity of many taxa within a functional group. This concept is similar to how individual plant species can influence rates of primary production in plant communities (Loreau et al., 2001; Loreau, 2010). Persistence of *Glomus* species under winter and growing-season warming may lead to maintenance of forest growth long-term, if the supply of soil nutrients (like nitrogen) can keep pace with plant photosynthetic demand.

N-cycling functional groups also shifted in relative abundance with CCASE treatments. We found that denitrifiers (i.e., taxa that complete the conversion of nitrate to N_2_ gas) generally declined in relative abundance under the *warmed* + *FTC* treatment (Figure 3). This decline was consistent across sampling time points during the growing season following winter soil freeze/thaw treatments (Figure 3), indicating that legacy effects of winter climate on total soil microbial activity (Sorensen et al., 2016, 2018; Väisänen et al., 2019) may be due to their effects on the composition of microbial functional groups driving that activity. These observations contrast previous reports that increased freeze/thaw cycles can induce anaerobic conditions in soil (Kay et al., 1981; De Bruijn et al., 2009), which are thought to favor anaerobic N cycling processes, such as denitrification. However, the *warmed* + *FTC* treatment had different impacts on each step in the denitrification process. Soil bacteria had reduced genetic ability to convert nitrous oxide (N_2_O) to N_2_ gas, but retained the capacity to reduce nitric oxide (NO) to N_2_O under colder soil temperatures (Supplementary Figure 6). The particular sensitivity of N_2_O-reducing bacteria to soil freezing may lead to long-term increases in N_2_O flux under year-round climate change, the same way that rates of N_2_O flux from soil often increase in early spring following snowmelt and soil thawing (Tatti et al., 2014).

### Hypothesis 3: Stress Tolerance-Decomposition Trait Trade-Offs Determine New Biogeochemical Functions Under Year-Round Climate Change

We hypothesized that soil freeze/thaw events in winter would slow C and N cycling in soil by selecting for taxa that trade-off C decomposition traits and nutrient uptake traits for stress (e.g., freezing, dessication) tolerance. While climate change manipulations did not significantly affect the abundance of most microbial genes hypothesized to confer tolerance to temperature extremes (e.g., 1,3-β-glucanase, trehalase, or RNA helicase), we found that the *warmed* + *FTC* treatment increased the genetic potential for stress tolerance in soil microbes in other ways. Specifically, numbers of genes coding for tyrosinase – an enzyme involved in melanin synthesis – were highest in the *warmed* + *FTC* treatment and lowest in the *warmed* treatment. Many microbes synthesize melanin in response to environmental stressors (Bell and Wheeler, 1986; Khajo et al., 2011), including declining temperatures, because melanin is an effective conductor of radiation and allows regulation of internal temperature (Cordero et al., 2018). In addition, genes involved in nicotinamide nucleotide coenzyme (NADH/NAD^+^) metabolism increased under the *warmed* + *FTC* treatment (Supplementary Figure 8). Levels of NAD^+^ change when cells experience stressors such as C starvation (Cantó et al., 2015), which may occur under winter climate change. At CCASE, the *warmed* + *FTC* treatment reduced soil microbial biomass, extracellular enzyme activity, and nutrient immobilization into microbial cells (Sorensen et al., 2018), perhaps because soil freeze/thaw alters the physical structure of soils, reducing diffusion of nutrients and water (Ostroumov and Siegert, 1996). We observed a negative correlation between NAD^+^/NADH metabolism and growth or reproduction capacity in the soil metagenomes across CCASE treatments (Figure 5), suggesting a trade-off between energy-based stress tolerance and fast rates of C and N metabolism in soil microbial communities. In the *warmed* + *FTC* treatment, losses of genes in the metagenome included those coding for proteins such as carotenoid oxygenase, proline racemace, and alpha-acetolactate decarboxylase (Supplementary Table 5), which are part of neurological processes and sensory organ and embryo development in higher organisms, but also function in various sensory and nutrient regulatory processes in microorganisms. For example, carotenoid oxygenase and retinal are used by bacteria, fungi, and archaea for light-driven ion transport and phototaxis (Spudich et al., 2000; Jung et al., 2003; Ruch et al., 2005) while proline racemace regulates intracellular and extracellular amino acid pools in E. coli (Goytia et al., 2007) and acetolactate decarboxylase generates low-molecular weight C compounds that are excreted from cells, often as fermentation products in response to stress (Goupil-Feuillerat et al., 1997). Lower capacity to regulate cellular functions like nutrient uptake and retention under the *warmed* + *FTC* treatment suggests that freeze/thaw cycles may select against microbes that proliferate via processing of labile nutrients. These observations corroborate the emerging theory that soil microbes can discriminate their C allocation to growth (yield), biogeochemical cycling (e.g., extracellular enzyme production), or stress tolerance (Malik et al., 2019). Interestingly, other types of stress tolerance emerged in the soil metagenome under warming alone. Genes involved in stress-activated signaling cascades in the cell (JNK cascade) were highest in the *warmed* treatment (Supplementary Figure 8). This signaling cascade can be activated by a number of factors, including hyperosmolarity and heat shock (Bogoyevitch and Kobe, 2006) and may be involved in activating heat shock proteins (Park and Liu, 2001).

Microbial taxa that persisted under the *warmed* + *FTC* treatment may also use other strategies to tolerate the stress of severe soil temperature fluctuations. Within the arbuscular mycorrhizal fungi, species in the genera *Entrophospora* and *Glomus* increased with the *warmed* + *FTC* treatment. These genera have been observed previously to tolerate freezing and drying and be more abundant in winter than other taxa (Klironomos et al., 2001). *Glomus* has been described as a freeze-tolerant genus (Addy et al., 1998), producing extraradical hyphae that can more deftly survive freezing and allow *Glomus* to colonize plants following thaw (Addy et al., 1994). In addition, *Glomus* species form large quantities of C-storage vesicles, potentially allowing them to be more freeze-resistant than arbuscular mycorrhizal fungi that do not form vesicles such as *Scutellospora calospora* (Klironomos, 2000). Another strategy is stress avoidance, where some microbial taxa might sporulate prolifically or have relatively aerodynamic spores that disperse to, and establish in, soils after stressful conditions have passed (Robinson, 2001). In contrast to other arbuscular mycorrhizal fungi that use hyphal extension to reach new plants, *Glomus* species vigorously produce spores and can maintain high infectivity (Addy et al., 1997) even if hyphal networks are disrupted (Smith and Read, 2010), as they may be during freezing and thawing of soil.

## Conclusion

Climate change across seasons (warming and more frequent freeze/thaw events) had a significant effect on the community structure of belowground microbial communities in a northern hardwood forest in New Hampshire, United States. We found evidence of a shift to microbial communities tolerant to freeze/thaw stress under year-round climate change, which could prove advantageous to the hardwood trees in the region, if it allows symbiotic relationships between soil microbes and plants to persist during projected changes in winter climate. However, these new soil microbial communities have unique biogeochemical traits that likely shape soil and ecosystem C and N cycling. Specifically, soil microbial communities that persist under growing season and winter climate change appear to trade-off the genetic capacity to grow, reproduce, and regulate uptake of labile nutrients for stress-tolerance. This observation indicates that soil freeze/thaw cycles selects against fast-growing microbes and those that promote C and N cycling in soil. However, we also found evidence that the genomic potential of soil microbes to drive soil N losses as N_2_O, rather than N_2_, may increase with winter climate change. Since N_2_O is a potent greenhouse gas, combined growing-season warming and winter freeze/thaw cycles may also feedback to climate warming in high-latitude ecosystems.

## Data Availability Statement

DNA sequence data are available online through the JGI IMG portal and in the JGI Genomes Online Database (GOLD Study ID Gs0114515).

## Author Contributions

JB and PT conceived of this contribution to the CCASE at Hubbard Brook Experimental Forest, which was originally developed and implemented by PG, RS-D, PS, and PT. The data collection was conducted by MG, RS-D, and PS and analysis was performed by JB. The manuscript was written primarily by MG and JB, while all authors contributed to editing the text.

## Conflict of Interest

The authors declare that the research was conducted in the absence of any commercial or financial relationships that could be construed as a potential conflict of interest.

## Footnotes

1 http://duk.sourceforge.net/

2 http://www.drive5.com/usearch/manual/uchime_algo.html
