# Supplementary material for: Soil Microbes Trade-Off Biogeochemical Cycling for Stress Tolerance Traits in Response to Year-Round Climate Change
Source: Front Microbiol. 2020 May 13;11:616. doi: 10.3389/fmicb.2020.00616 (PMC7238748; doi:10.3389/fmicb.2020.00616)
Supplement: Supplementary file 1 [file Data_Sheet_1.docx]

**Supplementary Materials**

**1 Supplementary Figures**

**Supplementary Figure 1.** Alpha diversity changes for fungi (A) and bacteria (B) across CCASE treatments.


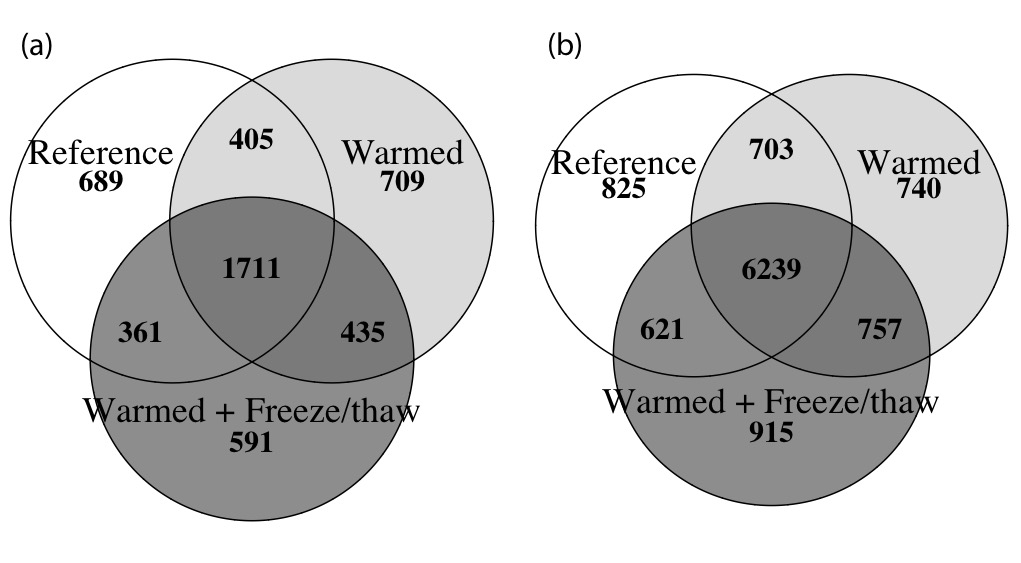


**Supplementary Figure 2.** Venn diagram of (a) fungal and (b) bacterial taxa distributions across CCASE treatments.

**Supplementary Figure 3.** Change in relative abundance of fungal phyla with CCASE treatments. Relative abundance of phyla was calculated as the summed total of each OTU within a phylum in a plot (n = 4 quadrants per plot). Change in phylum abundance in a plot was calculated at each time point relative to the pre-treatment time point (July 2013). Values for all OTUs in a phylum were then summed for each time point per plot.

**Supplementary Figure 4.** Change in relative abundance of bacterial phyla with CCASE treatments. Relative abundance of phyla was calculated as the summed total of each OTU within a phylum in a plot (n = 4 quadrants per plot). Change in phylum abundance in a plot was calculated at each time point relative to the pre-treatment time point (July 2013). Values for all OTUs in a phylum were then summed for each time point per plot.


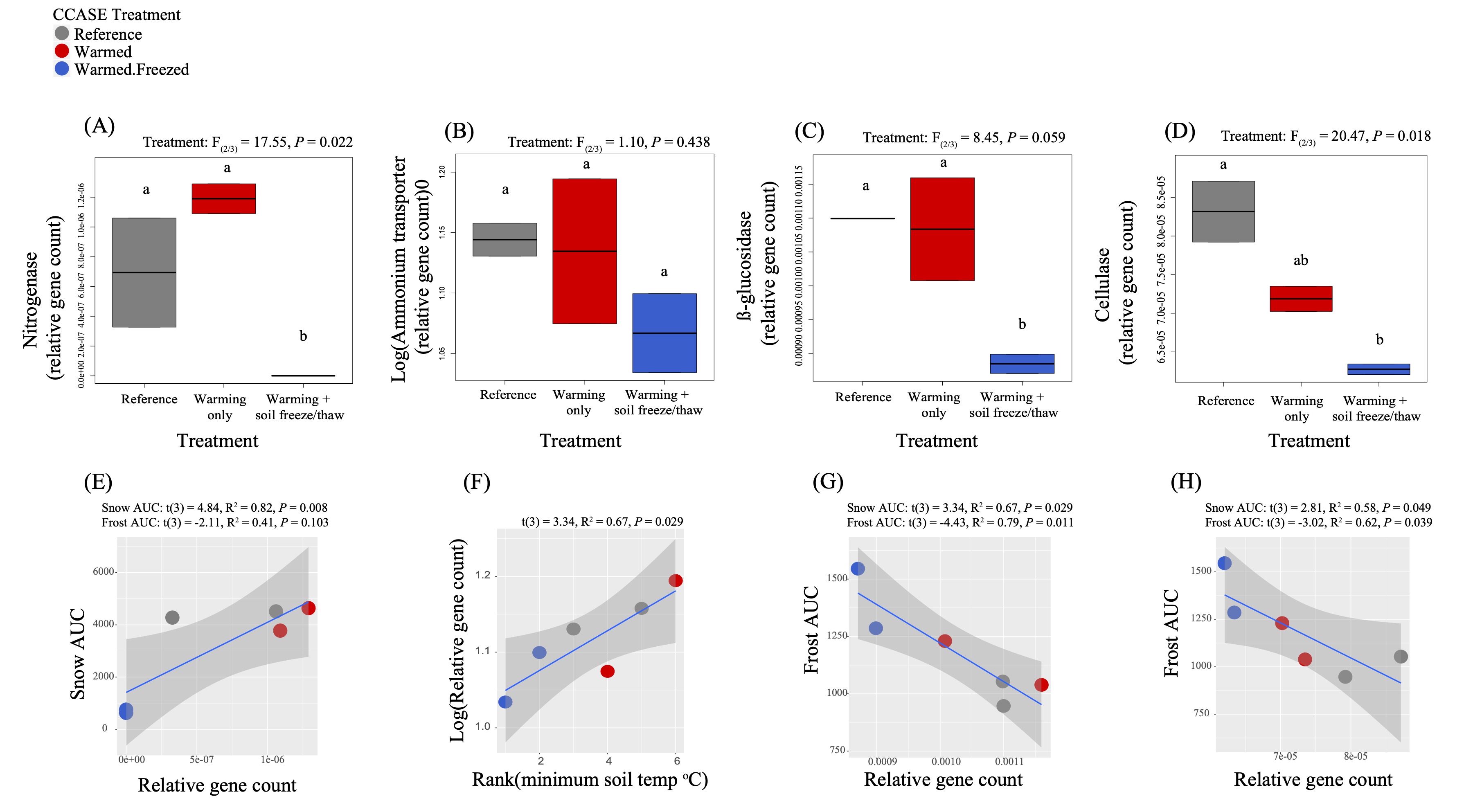


**Supplementary Figure 5.** Relative abundance of microbial genes within soil metagenomes across CCASE treatments. Genes coding for nitrogenase (panels A, E), ammonium transporters (panels B, F), B-glucosidase (panels C, G), and cellulase (panels D, H) are shown. Values are normalized gene counts per plot measured during peak plant biomass post-treatment (May 2014).


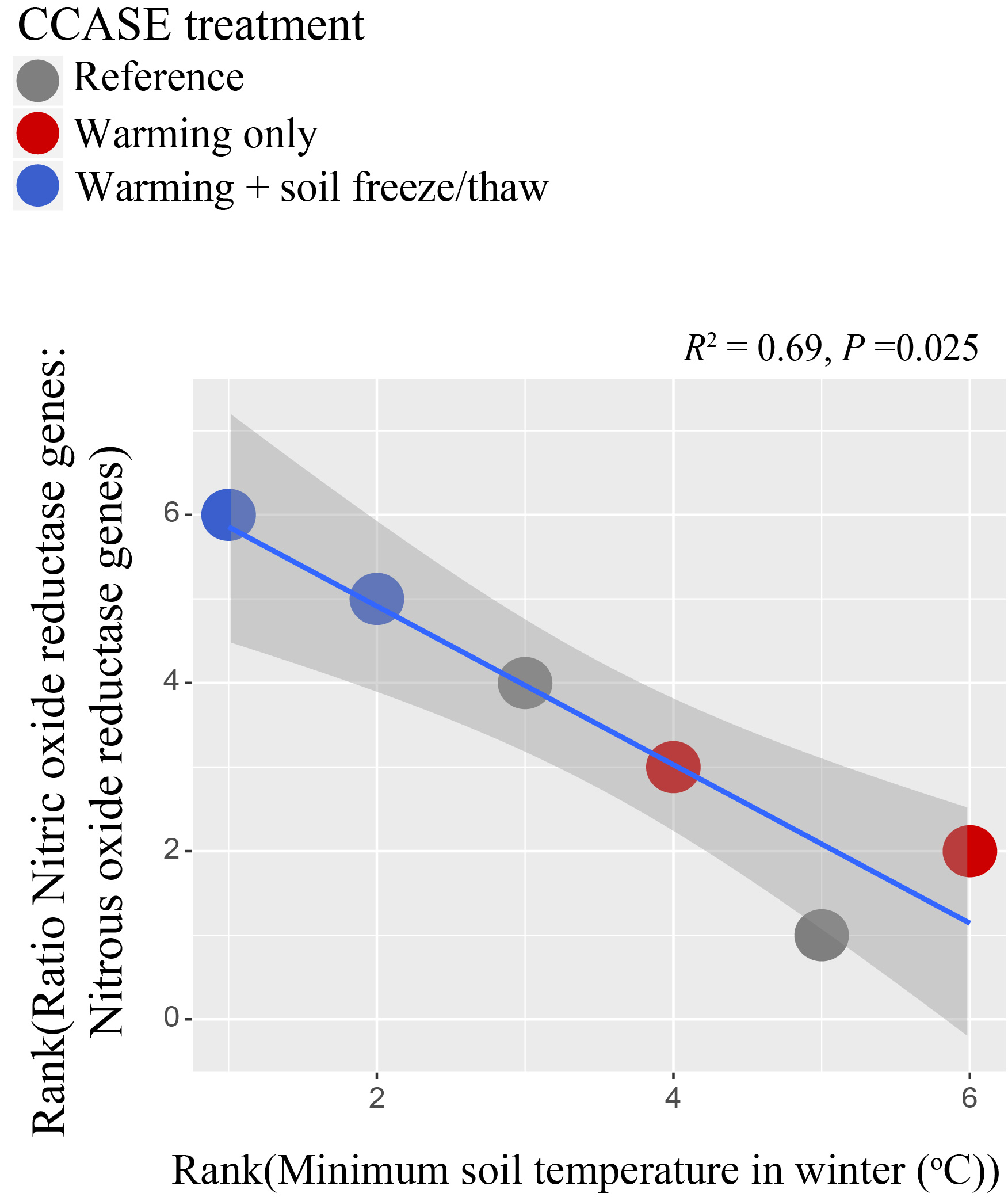


**Supplementary Figure 6.** Correlation between the ratio of nitric oxide reductase to nitrous oxide reductase genes in the soil metagenome and minimum temperatures in the top 10 cm of soil during winter. Metagenomes were sequenced from soil samples collected in May 2014 (full leaf-out), when biogeochemical differences among treatments were greatest (Sorensen et al., 2018).

**Supplementary Figure 7.** Shifts in the relative abundance of dominant taxa within microbial functional groups that change significantly with CCASE treatments (relative to pre-treatment). Functional groups include arbuscular mycorrhizal fungi (panels A, B), brown rot fungi (panels C, D), fungal parasites of animals (panels E, F), N fixers (panels G, H), Denitrifiers (panels I, J), and cellulose decomposer bacteria (panels K, L).

**
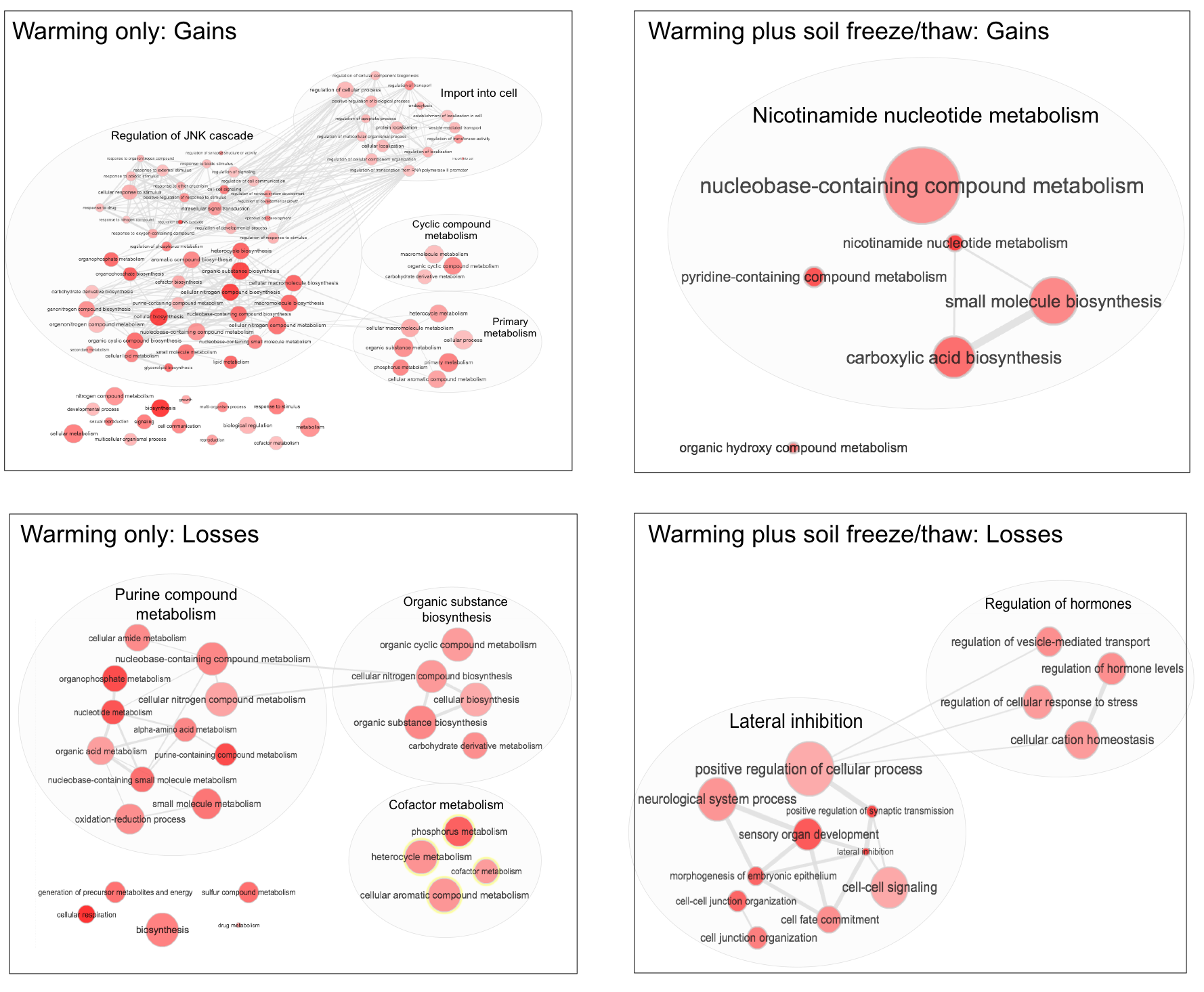
**

**Supplementary Figure 8.** Biochemical pathways significantly associated with CCASE treatments across soil fungi. Gene Ontology (GO) enrichment was calculated based on Pfam domains significantly positively (“Gains”) and negatively (“Losses”) correlated with each CCASE treatment (P < 0.1). Circle size represents the number of Pfam domains per GO category, with the larger circles representing the most domains. Circle shading indicates P value, with the smallest P values being indicated by the darkest shade of red. Gray lines indicate relatedness, and thickness of the line indicates degree of relatedness among GO terms.

**2 Supplementary Tables**

**Supplementary Table 1.** Environmental covariates measured at CCASE. Soil % N = Total percent soil N, Soil % C = Total percent soil C, MBN = Microbial Biomass N (µg N g soil-1), EOC = Extractable organic C (mM C), DIN = Dissolved inorganic N (µg N g soil-1), REL = Relative electrolyte leakage for root tissue (%), Photosynthesis = photosynthesis rate (μmolCO_2_ m^–2^ s^–1^) at peak biomass, Tree Basal Area = Basal area (M) of stems > 5 cm diameter in a plot. Soil temperature = Soil temperature (°C) at time of sampling, Soil moisture = Gravimetric soil water content (%) at time of sampling, Min Soil10 = Minimum winter soil temperature at 10 cm depth, Max frost = Maximum winter soil frost depth (cm), Max snow = Maximum snow depth (cm), Frost AUC = Area under the curve for winter frost depth over time (cm day), Snow AUC = Area under the curve for winter snow cover over time (cm day), FTC = Number of freeze/thaw cycles at 10 cm (-0.5 °C Threshold), DWF = Number of days with soil frost. Sampling time points include (1) Pre-budburst_post-snowmelt, (2) Budburst, (3) Full_leaf-out, (4) Full canopy, (5) Peak_plant_biomass, and (6) Beginning_of_senescence. Variables measured at the soil core level were measured on subsamples of the same soil cores taken for DNA extraction.

|  | **Sampling time point** | **Sampling level** | **Measurement and Calculation** | **Reference** |
| --- | --- | --- | --- | --- |
| **Soil % N** | 1-6 | Soil core | Individual values for each quadrant at each time point, based on flash combustion | Sorensen et al. 2018 |
| **Soil % C** | 1-6 | Soil core | Individual values for each quadrant at each time point, based on flash combustion | Sorensen et al. 2018 |
| **Soil pH** | 1-6 | Soil core | Individual values for each quadrant at each time point, based on 1:2 w/vol slurry in MilliQ H_2_O | Sorensen et al. 2018 |
| **MBN** | 1-6 | Soil core | Individual values for each quadrant, based on chloroform fumigation-extraction/alkaline persulfate digestion and colorimetric measurement of nitrate in K2SO4 extracts | Sorensen et al. 2018 |
| **EOC** | 2-6 | Soil core | Individual values for each quadrant at each time point, based on Mn(III)-pyrophosphate oxidation of K2SO4 extractions | Sorensen et al. 2018 |
| **DIN** | 2-6 | Soil core | Individual values for each quadrant at each time point, based on colorimetric measurement of nitrate and ammonium in KCl extracts | Sorensen et al. 2018 |
| **Amino acid-N** | 2-6 | Soil core | Individual values for each quadrant at each time point, based on OPAME method of quantification on TCA/toluene treated sodium acetate extracts | Sorensen et al. 2018 |
| **Soil CO_2_ flux** | 1-6 | Soil core | Individual values for each quadrant at each time point, based on periodic measurements during 28-day lab incubation using EGM-4 | Sorensen et al. 2018 |
| **REL** | 1-6 | Soil core | Individual values for each quadrant at Peak Biomass, based on solution conductivity following detergent extraction of roots pre- and post-heat killing | Sanders-Demott et al. 2018 |
| **Photosynthesis** | 5 | Plot | Single value per plot at Peak Biomass, based on average LiCOR measurements of 5 technical reps of 3 leaves from each of 4 target trees | --- |
| **Tree Basal Area** | 1 | Plot | Single value per plot at Peak Biomass, based on DBH measurement of >5 cm diameter trees | Templer et al. 2017 |
| **Soil temperature** | 1-6 | Soil core | Measured throughout the year using thermistors buried at 10 cm (n = 6 per plot in heated plots and n = 4 per plot in reference plots) | Sorensen et al. 2018 |
| **Soil moisture** | 1-6 | Soil core | Individual values for each quadrant at each time point, based on gravimetric water content | Sorensen et al. 2018 |
| **Min Soil10** | 2013/2014 Winter | Plot | Minimum for each plot in the winter season using the average of all half-hourly temperature sensors data | Sanders-Demott et al. 2018 |
| **Max frost** | 2013/2014 Winter | Plot | Maximum for each plot using the soil frost data collected from frost tubes, sampled every 2-6 days | Sanders-Demott et al. 2018 |
| **Max Snow** | 2013/2014 Winter | Plot | Maximum for each plot using the snow depth data collected from frost tubes, sampled every 2-6 days | Sanders-Demott et al. 2018 |
| **Frost AUC** | 2013/2014 Winter | Quadrant | Single value per plot for all time points, AUC for each plot using the soil frost data | Sanders-Demott et al. 2018 |
| **Snow AUC** | 2013/2014 Winter | Quadrant | Single value per plot for all time points, AUC for each plot using the snow depth data | Sanders-Demott et al. 2018 |
| **FTC** | 2013/2014 Winter | Plot | Single value per plot for all time points, based on the 10 cm temperature sensors | Sanders-Demott et al. 2018 |
| **DWF** | 2013/2014 Winter | Plot | Single value per plot for all time points, the dates where the depth of frost was greater than 0cm | Sanders-Demott et al. 2018 |

**Supplementary Table 2a**. Correlation statistics between environmental variables and soil microbial community composition. Statistics are generated from single factor perMANOVA analysis.

|  |  |  | **Fungal community composition** | | | **Prokaryotic community composition** | | |
| --- | --- | --- | --- | --- | --- | --- | --- | --- |
| Variable name | Variable description | df | R^2^ | F statistic | P value | R^2^ | F statistic | P value |
| Single factor  CCASE Treatment  Soil temperature  Max frost  Max snow  frost AUC  snow AUC  Days With Frost  Min.Soil10  FTC  Total tree basal area  REL  Soil % N  Soil % C  EOC  Soil moisture  DIN  Soil pH  MBN  Date sampled  Spatial distance | Experimental Treatment  Soil temperature (°C) at time of sampling  Maximum winter soil frost depth (cm)  Maximum snow depth (cm)  Area under the curve for winter frost depth over time (cm day)  Area under the curve for winter snow cover over time (cm day)  Number of days with soil frost  Minimum winter soil temperature at 10 cm depth  Number of freeze/thaw cycles at 10 cm (-0.5 °C Threshold)  Basal area (M) of stems > 5 cm diameter in a plot  Relative electrolyte leakage for root tissue (%)  Total percent soil N  Total percent soil C  Extractable organic C (mM C)  Gravimetric soil water content (%)  Dissolved inorganic N (µg N g soil-1)  pH in top 10 cm soil  Microbial Biomass N (µg N g soil-1)  Sampling period of the 2014 growing season (e.g. “budburst”)  Distance between quadrants (m) | 2  1  1  1  1  1  1  1  1  1  1  1  1  1  1  1  1  1  5  -- | 0.036  0.018  0.018  0.020  0.015  0.022  0.019  0.021  0.022  0.020  0.010  0.020  0.021  0.012  0.020  0.015  0.016  0.023  0.069  0.024 | 2.14  2.14  2.06  2.40  1.77  2.53  2.18  2.49  2.56  2.42  1.19  2.35  2.52  1.44  2.36  1.41  1.87  2.67  1.67  164.61 | **0.001**  **0.001**  **0.001**  **0.001**  **0.001**  **0.001**  **0.001**  **0.001**  **0.001**  **0.001**  0.098  **0.001**  **0.001**  **0.006**  **0.001**  **0.016**  **0.001**  **0.001**  **0.001**  **<0.0001** | 0.040  0.035  0.015  0.020  0.008  0.020  0.023  0.022  0.021  0.035  0.009  0.073  0.081  0.030  0.038  0.026  0.028  0.083  0.126  0.014 | 2.29  4.04  1.69  2.26  0.91  2.27  2.58  2.54  2.41  4.05  0.98  8.74  9.88  3.42  4.43  2.40  3.18  9.75  3.27  89.58 | **0.001**  **0.001**  **0.040**  **0.008**  0.535  **0.007**  **0.003**  **0.001**  **0.003**  **0.001**  0.437  **0.001**  **0.001**  **0.001**  **0.001**  **0.003**  **0.001**  **0.001**  **0.001**  **0.003** |

**Supplementary Table 2b**. Multiple regression correlation statistics between environmental variables and soil microbial community composition. Statistics are generated from multiple regression on matrices (MRM, in the case of spatial distance).

|  |  | **Fungal community composition** | | | **Prokaryotic community composition** | | |
| --- | --- | --- | --- | --- | --- | --- | --- |
| Variable name | Variable description | R^2^ | F statistic | P value | R^2^ | F statistic | P value |
| Multiple regression  snow AUC  Max snow  Min.Soil10  FTC  Days With Frost  Total tree basal area  Soil % C  Soil % N  Soil moisture  Soil temperature  EOC  DIN  MBN  Soil pH  Date sampled  Spatial distance | Area under the curve for winter snow cover over time (cm day)  Maximum snow depth (cm)  Minimum winter soil temperature at 10 cm depth  Number of freeze/thaw cycles at 10 cm (-0.5 °C Threshold)  Number of days with soil frost  Basal area (M) of stems > 5 cm diameter in a plot  Total percent soil C  Total percent soil N  Gravimetric soil water content (%)  Soil temperature (°C) at time of sampling  Extractable organic C (mM C)  Dissolved inorganic N (µg N g soil-1)  Microbial Biomass N (µg N g soil-1)  pH in top 10 cm soil  Sampling period of the 2014 growing season (e.g. “budburst”)  Distance between quadrants (m) | 0.058 | 35.44 | 0.0002  0.922  0.255  0.107  **0.049**  -------  **0.001**  0.258  0.877  0.159  -------  -------  -------  0.999  -------  **0.004**  **0.0006** | 0.081 | 25.44 | 0.004  **0.018**  0.087  0.259  0.313  0.369  **0.022**  0.559  0.727  0.197  0.752  0.102  0.550  0.768  **0.045**  **0.0008**  ------- |

**Supplementary Table 3**. Specific microbial genes thought to vary with year-round climate change in soil metagenomes. Genes were matched to microbial functions and gene names via KEGG orthologies and the Pfam database.

| Protein name | Function | Gene(s) |
| --- | --- | --- |
| 1,3-β-glucan synthase  Trehalase  RNA helicase  Polyketide synthase  Tyrosinase  Laccase  β-glucosidase  Cellobiosidase  Endoglucanase  Cellulase  Nitrogenase  Ammonia monooxygenase  Hydroxylamine dehydrogenase  Nitrate reductase  Nitrite reductase  Nitrite reductase (NO-forming)  Nitric oxide reductase  Nitrous oxide reductase | Low temperature tolerance/stress response; antifreeze production  Low temperature tolerance/stress response; antifreeze production  Low temperature tolerance/stress response; RNA folding  Stress response; melanin production  Stress response; melanin production  Stress response; melanin production  Cellulose decomposition  Cellulose decomposition  Cellulose decomposition  Cellulose decomposition  Nitrogen fixation  Nitrification  Nitrification  Nitrification/Dissimilatory nitrate reduction; nitrate 🡪 nitrite  Dissimilatory nitrate reduction; nitrite 🡪 ammonia  Denitrification; nitrite 🡪 NO  Denitrification; NO 🡪 N_2_O  Denitrification; N_2_O 🡪 N_2_ | FKS1  NTH1  RhlE/ RhlB/ SNF2/ SUPV3L1/SUV3/DeaD/ HelY/ DHX37/DHR1/ DHX57/ DHX8/PRP22/ DOB1/ Mtr4  PKS1  TYR  AA1  GH1/3  GH5/7  AA9  GH 9/10/12/44/43  nifD/anfG/vnfD  pmoa-amoA  hao  narG/narZ/nxrA/napA  nrfA/nirB  nirK/nirS  norB  nosZ |

**Supplementary Table 4.** Pearson correlation coefficients for soil variables measured at each soil sampling point (n = 127). Soil % N = Total percent soil N, Soil % C = Total percent soil C, MBN = Microbial Biomass N (µg N g soil-1), EOC = Extractable organic C (mM C), DIN = Dissolved inorganic N (µg N g soil-1), REL = Relative electrolyte leakage for root tissue (%), Photosynthesis = photosynthesis rate (μmolCO_2_ m^–2^ s^–1^) at peak biomass, Tree Basal Area = Basal area (M) of stems > 5 cm diameter in a plot. Asterisks represent significance of correlation (*****P* < 0.0001, ****P* < 0.001, ***P* < 0.01, **P* < 0.05).

|  | **Soil % N** | **Soil % C** | **Soil pH** | **MBN** | **EOC** | **DIN** | **Amino acid-N** | **Soil CO_2_ flux** | **REL** | **Photosynthesis** | **Tree Basal Area** |
| --- | --- | --- | --- | --- | --- | --- | --- | --- | --- | --- | --- |
| **Soil % N** | 1.00 | 0.97**** | 0.08 | 0.90**** | 0.37 | 0.71*** | 0.56** | 0.36 | 0.32 | -0.09 | -0.19 |
| **Soil % C** | 0.97**** | 1.00 | -0.03 | 0.88**** | 0.43* | 0.74*** | 0.55* | 0.43 | 0.32 | -0.13 | -0.21 |
| **Soil pH** | 0.08 | -0.03 | 1.00 | 0.03 | 0.04 | 0.11 | -0.03 | 0.00 | -0.14 | 0.20 | 0.28 |
| **MBN** | 0.90**** | 0.88**** | 0.03 | 1.00 | 0.30 | 0.53* | 0.59** | 0.49* | 0.18 | -0.15 | -0.08 |
| **EOC** | 0.37 | 0.43* | 0.04 | 0.30 | 1.00 | 0.60** | 0.19 | 0.29 | 0.05 | -0.39 | 0.34 |
| **DIN** | 0.71*** | 0.74*** | 0.11 | 0.53* | 0.60** | 1.00 | 0.34 | 0.26 | 0.22 | -0.08 | -0.19 |
| **Amino acid-N** | 0.56** | 0.55* | -0.03 | 0.59** | 0.19 | 0.34 | 1.00 | 0.08 | -0.06 | -0.37 | 0.05 |
| **Soil CO_2_ flux** | 0.36 | 0.43 | 0.00 | 0.49* | 0.29 | 0.26 | 0.08 | 1.00 | 0.15 | -0.13 | 0.17 |
| **REL** | 0.32 | 0.32 | -0.14 | 0.18 | 0.05 | 0.22 | -0.06 | 0.15 | 1.00 | 0.24 | -0.29 |
| **Photosynthesis** | -0.09 | -0.13 | 0.20 | -0.15 | -0.39 | -0.08 | -0.37 | -0.13 | 0.24 | 1.00 | -0.74*** |
| **Tree Basal Area** | -0.19 | -0.21 | 0.28 | -0.08 | 0.34 | -0.19 | 0.05 | 0.17 | -0.29 | -0.74*** | 1.00 |

**Supplementary Table 5.** Pearson correlation coefficients for climate variables. Soil temperature = Soil temperature (°C) at time of sampling, Soil moisture = Gravimetric soil water content (%) at time of sampling, Min Soil10 = Minimum winter soil temperature at 10 cm depth, Max frost = Maximum winter soil frost depth (cm), Max snow = Maximum snow depth (cm), Frost AUC = Area under the curve for winter frost depth over time (cm day), Snow AUC = Area under the curve for winter snow cover over time (cm day), FTC = Number of freeze/thaw cycles at 10 cm (-0.5 °C Threshold), DWF = Number of days with soil frost. Asterisks represent significance of correlation (*****P* < 0.0001, ****P* < 0.001, ***P* < 0.01, **P* < 0.05).

|  | **Soil temperature** | **Soil moisture** | **Min Soil10** | **Max frost** | **Max Snow** | **Frost AUC** | **Snow AUC** | **FTC** | **DWF** |
| --- | --- | --- | --- | --- | --- | --- | --- | --- | --- |
| **Soil temperature** | 1.00 | -0.40**** | -0.19* | 0.06 | -0.25** | 0.11 | -0.24** | 0.21* | -0.19* |
| **Soil moisture** | -0.40**** | 1.00 | 0.21* | 0.02 | 0.19* | -0.05 | 0.19* | -0.19* | 0.30*** |
| **Min Soil10** | -0.19* | 0.21* | 1.00 | -0.60**** | 0.85**** | -0.30*** | 0.94**** | -0.98**** | 0.89**** |
| **Max frost** | 0.06 | 0.02 | -0.60**** | 1.00 | -0.46**** | 0.77**** | -0.59**** | 0.62**** | -0.42**** |
| **Max Snow** | -0.25** | 0.19* | 0.85**** | -0.46**** | 1.00 | -0.17* | 0.94**** | -0.90**** | 0.64**** |
| **Frost AUC** | 0.11 | -0.05 | -0.30**** | 0.77**** | -0.17* | 1.00 | -0.30*** | 0.31*** | -0.25** |
| **Snow AUC** | -0.24** | 0.19* | 0.94**** | -0.59**** | 0.94**** | -0.30*** | 1.00 | -0.97**** | 0.75**** |
| **FTC** | 0.21* | -0.19* | -0.98**** | 0.62**** | -0.90**** | 0.31*** | -0.97**** | 1.00 | -0.80**** |
| **DWF** | -0.19* | 0.30*** | 0.89**** | -0.42**** | 0.64**** | -0.25** | 0.75**** | -0.80**** | 1.00 |
